# Atypical leg symptoms: does routine measurement of the ankle brachial pressure index (ABPI) in primary care benefit patients?

**DOI:** 10.1080/17571472.2015.1082345

**Published:** 2015-09-28

**Authors:** Christine Oesterling, Amun Kalia, Thomas Chetcuti, Steven Walker

**Affiliations:** ^a^ Eastmead Surgery, Ealing CCG. Greenford, UK; ^b^ Kingston Hospital NHS Trust, Kingston upon Thames, UK; ^c^ Ridgeway Surgery, Harrow CCG, North Harrow, UK; ^d^ St Giles Medical, London, UK

**Keywords:** Atypical leg symptoms, leg pain, Doppler assessment, ankle brachial pressure index (ABPI), primary care

## Abstract

**Background:**

Managing patients with atypical leg symptoms in primary care can be problematic. Determining the ankle brachial pressure index (ABPI) may be readily performed to help diagnose peripheral arterial disease, but is often omitted where signs and symptoms are unclear.

**Question:**

Does routine measurement of ABPI in patients with atypical leg symptoms aid management increase satisfaction and safely reduce hospital referral?

**Methodology:**

Patients with atypical leg symptoms but no skin changes or neurological symptoms underwent clinical review and Doppler ABPI measurement (suspicious finding ≤ 1.0). Testing was performed by the same doctor (study period: 30 months). Patient outcomes were determined from practice records, hospital letters and a telephone survey.

**Results:**

The study comprised 35 consecutive patients (males: *N* = 15), mean age 64 years (range: 39–88). Presentation included pain, cold feet, cramps, irritation and concerns regarding circulation. Prior to ABPI measurement, referral was considered necessary in 10, not required in 22 and unclear in 3. ABPI changed the referral decision in 10 (29%) and confirmed the decision in 25 (71%). During the study, 10 (29%) patients were referred (9 vascular, 1 neurology). Amongst the vascular referrals, significant peripheral arterial disease has been confirmed in six patients. A further two patients are under review and one did not attend. To date, lack of referral in patients with atypical leg symptoms but a normal ABPI has not increased morbidity. Current status was assessed by telephone review in 16/35 (46% contact rate; mean 18 months, range 2–28). Fifteen patients (94%) appreciated that their symptoms had been quickly and conveniently assessed, 8/11 (73%) with a normal ABPI were reassured by their result and in 8/11 symptoms have resolved.

**Discussion/Conclusion:**

APBI conveniently aids management of atypical leg symptoms by detecting unexpected peripheral arterial disease, avoids /confirms the need for referral, reassures patients and guides reassessment. This study suggests ABPI should be used more widely.

## Key messages

• Determining the ankle-brachial pressure index in patients with atypical leg symptoms helps decision-making.
• Overall referral numbers were not reduced by testing, but were more appropriately targeted.
• In this study, non-referral of patients with atypical leg symptoms but a normal ankle-brachial pressure index has to date not resulted in increased morbidity.
• Determining the ankle-brachial pressure index aids management of atypical leg symptoms in primary care by:
  ○ Detecting unexpected peripheral arterial disease.
  ○ Avoiding or confirming the need for referral.
  ○ Directing further questioning and examination where signs and symptoms were initially unclear.
  ○ Clarifying the clinical situation amongst those with multiple pathologies or where communication is difficult.
  ○ Reassuring patients and increasing satisfaction.

## Why this matters to me

In our experience, patients presenting with atypical leg symptoms, where the diagnosis is unclear, is a relatively common problem. Deciding how best to manage such patients in primary care can be problematic. The National Institute for Clinical Excellence recommends measuring the ankle-brachial pressure index where peripheral arterial disease is suspected. In a busy practice, peripheral arterial disease is often not considered when the history and examination are equivocal. Such patients are sometimes referred unnecessarily, whilst others may miss the opportunity for early treatment. Could a simple test help? This limited study suggests that routine measurement of the ankle-brachial pressure index is easy and quick to perform, is appreciated by patients, aids decision-making and can safely avoid/confirm the need for referral. We recommend routine testing for all patients presenting in primary care with atypical leg symptoms.

## Background

Lower limb symptoms are prevalent, especially in older adults.[1] The aetiology is generally related to musculoskeletal, neurogenic or vascular disease.[2] Classical features such as limited, painful joint movement, discomfort on stretching the sciatica nerve, varicose veins, ulceration or intermittent claudication point to a likely diagnosis and an appropriate course of action. Unfortunately, disease processes and symptomatology do not always follow classical patterns. A situation encountered relatively commonly in primary care is a patient with symptoms in their legs of unclear origin. Deciding how best to manage atypical leg symptoms can be problematic, especially where multiple pathologies are suspected. In a busy surgery, it may not occur to the doctors that the patient is suffering from peripheral arterial disease. Many such patients will undergo fruitless investigations, attempts at treatment and unnecessary referrals.[3] Conversely, others with treatable disease may end up being labelled as having medically unexplained symptoms [4] or suffering from a somatic symptom disorder.[5] Clinical experience suggests that affected patients in primary care could benefit from a reliable diagnostic tool to aid management.

Peripheral arterial disease is a common problem which often presents with atypical leg symptoms and is increasingly treatable. It is claimed that 12–14% of the general population are affected, notably those over 70 years of age.[6] Patients and their doctors are often unaware of the presence of peripheral arterial disease, with only 10% of subjects experiencing the typical features of intermittent claudication (limb pain on exercise relieved by rest).[7] Aetiological factors include smoking, diabetes, hypertension and dyslipidaemia.[8] Detection benefits patients by enabling treatment to prevent critical ischaemia and possible amputation, as well as facilitating intervention and lifestyle changes at an earlier stage when management may be more successful.[9] Peripheral arterial disease is also a marker of systemic atherosclerosis and cardiovascular risk.[10] Around one third of patients with peripheral arterial disease die within five years of their diagnosis, primarily due to a heart attack or stroke.[11]

Determining the ankle-brachial pressure index (ABPI) is a simple test for the diagnosis of peripheral arterial disease.[12] It is widely used by nursing staff in leg ulcer clinics to manage venous ulceration and confirm that compression bandaging can be safely applied without endangering the arterial supply.[13] By comparison, determining the ABPI is less commonly employed by primary care physicians in the absence of skin changes or where lower limb symptoms are unclear.[11] This may be due to unfamiliarity, time pressure or a belief that because it is a relatively cheap and unsophisticated test its benefits must therefore be limited. Their views may have been influenced by a number publications from vascular centres suggesting that ABPI measurements have been superseded.[6]

In the UK, there is pressure to manage more patients in the community. Most GPs are experiencing higher workloads and are required to manage increasingly complex patients during a standard 10-min consultation. Against this background could routine determination of the ABPI be of benefit in primary care for patients with atypical leg symptoms despite the reservations expressed above? Information is limited. A paper by Hooi and colleagues from Maastricht concluded that ABPI measurement can be a useful supplementary test in ambiguous situations.[14]

The aim of the present study was to determine whether routine measurement of ABPI in patients presenting with atypical leg symptoms can safely aid management, increase satisfaction and reduce hospital referral rates.

## Methodology

The study population comprises a consecutive series of patients attending a busy NHS teaching practice with leg symptoms where the initial history and examination did not point to cause or a rational management plan and who subsequently underwent ABPI measurement. The practice is located in West London and looks after a mix of racial groups for whom English is frequently their second language. Patients are often only registered for short periods.

Patients were seen over a 30-month period by a mixture of senior staff and trainees. During the initial consultation, the doctor sought to identify a possible musculoskeletal, neurological or vascular cause for their leg symptoms. Clinical examination included feeling for foot pulses, observing the lower limb for skin changes and testing for any gross neurological abnormalities. Patients either underwent ABPI measurement during the same attendance or within one week. Testing was always performed by the same doctor (CO) using the technique in Textbox 1. Patients were excluded from this study where the history and examination pointed to a likely diagnosis and in whom the next steps in terms of investigations, treatment or specialist referral were clear.

Textbox 1. **How to measure the ABPI.**

**Table:** 

*Equipment:*
NICE recommend using a hand-held Doppler probe with a frequency of 7–10 mHz.[16] These are widely available from medical supply companies or via the internet. The cost of a standard vascular Doppler unit with a 8 mHz probe is around £50–£160.
Manual sphygmomanometer with a cuff that fits comfortably around the patient’s limb. Ideally, the bladder width should be 40% and length 80% of the arm circumference, too small and an abnormally high value is obtained. NICE consider that a manual unit is more reliable than an automated oscillometric device.[16]
Ultrasound gel.
*Positioning:*
The patient lies comfortably in the supine position. If this is not possible e.g. patient is in a wheelchair, then this should be recorded, as the ‘true’ ankle pressure is likely to be lower. Where critical, a correction factor may be applied.[16]
Allow a period of rest sufficient for the blood pressure to normalise.
*Brachial systolic pressure:*
Place the cuff around the upper arm. Feel for the brachial pulse. Apply gel over the artery.
With the probe held at an angle of 45–60°, adjust until arterial sounds are heard.
Inflate the cuff until mmHg above the point where arterial sounds disappear. Slowly deflate the cuff in small intervals until arterial sounds reappear. This is the brachial systolic pressure.
Repeat on other arm. Use the higher value to calculate the ABPI
*Ankle systolic pressure:*
Place cuff around lower leg, 5 cm above the medial malleolus. Feel for the posterior tibial and dorsalis pedis arteries. Apply gel.
Repeat the steps above in both legs. The ankle pressure is the highest recorded value for that leg.
Calculating the ABPI:
The ABPI for each leg is calculated as the highest detected ankle pressure divided by the highest systolic pressure in either arm e.g.

*An example:*
With the patient in the supine position on the couch if the highest systolic pressure is found to be 132 mmHg in the right arm and the highest systolic pressure in the left leg is measured as 90 mmHg over the dorsalis pedis artery (the posterior tibial could not be found), then the calculation for the left leg is

In the right leg both arteries could be detected, with the highest value of 120 mmHg being measured over the posterior tibial artery. The calculation is:

The patient is likely to have moderate arterial disease in their left leg and may also be suffering a degree of obstruction in the opposite leg. A vascular referral is likely to be justified.
*Interpretation of resting ABPI*[6]
>1.4 Suggests non-compressible arteries due to calcification
>1.0 Arterial disease unlikely
0.81–1.0 No significant or only mild arterial disease
0.5–0.80 Moderate arterial disease
<0.5 Critical limb ischaemia

In this study, an ABPI of ≤1.0 was considered suspicious and worthy of further consideration.

Patient outcomes were determined from a review of the practice records, hospital letters and a telephone survey conducted by one doctor (AK).

## Results

The study population comprised 35 consecutive patients (males: *N* = 15, mean age 64 years; range: 39–88) with atypical leg symptoms who underwent ABPI measurement. Presenting complaints included atypical pain, ‘cold feet’, ‘cramps’, ‘irritations’ and concerns regarding ‘poor circulation’.

ABPI proved relatively simple and quick to perform. Measurement of ABPI took a mean of 10 min (range 5–20 min) and, despite some discomfort, was generally well tolerated. Delay was mostly caused by the need for limb exposure, patient positioning on the examination couch and time for blood pressure to return to resting levels.

The ABPI in one or both legs was found to be ≤1.0 in 10 patients, of whom eight were referred to a vascular specialist for urgent review, one was referred after a trial of lifestyle modification and one with borderline results and a cardiac history is being managed conservatively.

Only one patient with a normal ABPI has so far been referred. A 78-year-old female presented with leg pain on exertion and a previous history of laryngeal problems diagnosed by an ENT specialist as ‘silent reflux’. Her ABPI in both legs was 1.15. She was referred to a neurologist who subsequently diagnosed motor neuron disease.

Of the nine patients reviewed by a vascular specialist, clinically important peripheral arterial disease has so far been confirmed by angiography in six patients, of whom five underwent endarterectomy or angioplasty (± stent insertion). A further two patients are under review. One patient, a 78-year-old lady with atypical leg pain and an unexpectedly low ABPI (right 0.85, left 0.76) did not attend her vascular appointment. Her symptoms have been much improved by low-dose amitriptyline.

**Figure 1.**
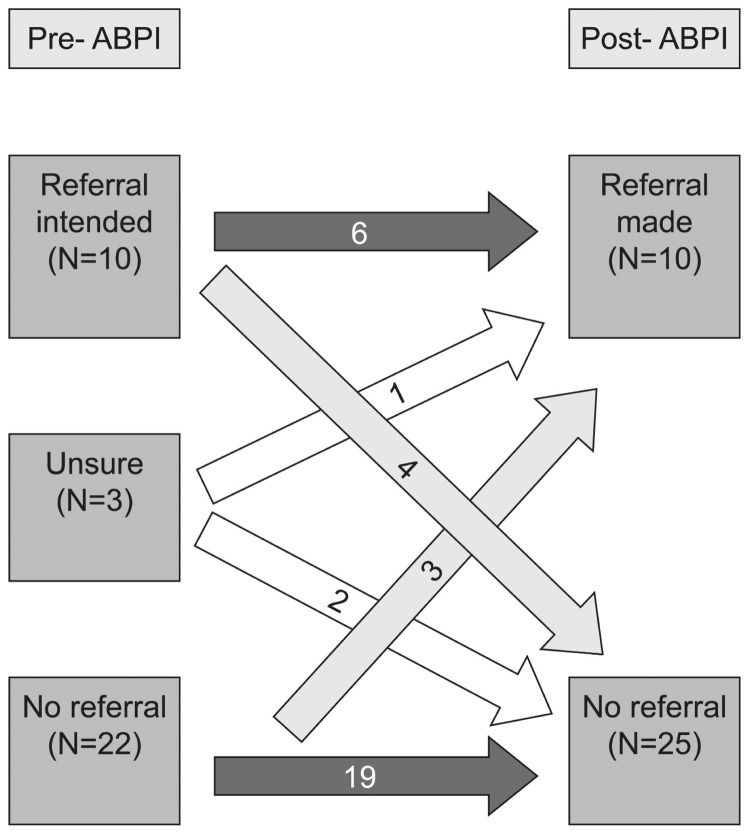
How determining the ABPI influenced the referral decision: on the left are depicted the number of patients for whom referral was planned, not planned or where the decision was unclear. On the right are shown the number of patients referred or not referred after ABPI. The arrows show changes between groups (*N* = 30).

In this study, overall referral numbers were not reduced by ABPI measurement but those sent to a specialist were more appropriately targeted. Prior to ABPI measurement, referral was considered to be necessary in 10 patients, not required in 22 and unclear in 3. ABPI changed the referral decision in 10 (29%) and confirmed the decision in 25 (71%) (Figure 1). To date, no patient with atypical leg symptoms and normal ABPI measurements has to our knowledge suffered increased morbidity related to non-or delayed referral.

For individual patients, testing did make a difference. It helped direct further questioning/examination whenever an unexpected result was encountered. It also clarified the clinical situation in the presence of multiple pathologies. Two case histories are presented for illustration (Textboxes 2 and 3).

Textbox 2. **Case history 1 – Referral not planned but decision changed by ABPI result.**

**Table:** 

A 79-year-old Asian male presented with sudden onset of constant pain in his right big toe with no associated features. He had previously undergone a coronary artery bypass 13 years earlier, but there was nothing to suggest peripheral arterial disease at this presentation and vascular referral was not planned. Uric acid measurement and foot X-ray were normal. ABPI on the right was 0.76 and 1.3 on the left. Angiography subsequently showed a long occlusion of his right superficial femoral artery.

Textbox 3. **Case history 2 – Referral planned but decision changed by ABPI result.**

**Table:** 

A 71-year-old diabetic with heart failure and polyneuropathy presented with burning pain in both feet and ankles. Vascular referral was originally planned, but the decision was reversed after ABPI measurement (right 1.12, left 1.12). Telephone review at 21 months found that her original pain had gone. She was pleased that her symptoms were quickly and conveniently dealt with without the need to attend hospital.

Current patient status was assessed by telephone review in 16/35 (46% contact rate) at a mean interval of 18 months (range 2–28). Fifteen patients (94%) appreciated that their symptoms had been quickly and conveniently assessed without the need to attend a hospital clinic. Eight of 11 (73%) patients with normal ABPI values were reassured by their test results and in 8/11 symptoms have since resolved.

## Discussion

Approximately 1 in 20 general practice consultations result in referral to another service. A report by the King’s Fund [15] suggests that a significant proportion of referrals made may not be clinically necessary. Further, there is scope for improvement in the quality of referrals and in helping patients get to their correct destination.[15] In this study of patients with atypical leg symptoms determining the ABPI altered the management in nearly one third (29%) by aiding more appropriate referral, but it did not reduce overall referral numbers. Had the initial decision been based solely on a brief history and examination, then 4 out of 10 patients would have been referred unnecessarily. Similarly, 3 of 22 patients, in whom the decision was initially made not to refer, received potentially life or limb-saving treatment following ABPI determination. The situation was clarified in a further three patients where the decision to refer was initially unclear. Available information suggests that non-referral of patients with normal ABPI measurements has not so far increased morbidity.

ABPI is not the answer to everything in patients with atypical leg symptoms, but does help management by identifying one important causation, namely, reduced limb perfusion. Consequently, the National Institute for Clinical Excellence (NICE) recommends assessing the ABPI whenever peripheral arterial disease is suspected.[16] Testing also addresses a number of patient aspirations which include receiving a good treatment experience, greater responsiveness, better co-ordination of care and the provision of extra services.[15] Further, many patients do not relish having to attend a hospital outpatient department.[17] It is therefore unsurprising that participants appreciated the extra effort being taken to resolve their problems in familiar surroundings. Also, a high proportion felt reassured by the test, possibly in part due to the longer consultation which afforded a greater opportunity to interact with their doctor.

As discussed, symptoms are a poor guide to peripheral arterial disease.[6] Even if the GP has limited knowledge regarding leg problems, determining the ABPI is a quick, low cost and easy-to-learn first step. It is particularly useful in the presence of multiple pathologies and buys time for reflection and further questioning. Whilst testing prolongs the consultation, there may be potential savings to the NHS by reducing unnecessary referrals and getting the patient to the right specialist early in their disease process.

Using a hand-held Doppler and manual sphygmomanometer to determine ABPI is the most common measurement technique in primary care.[16] Are there other reliable alternatives? Using a diagnostic aid such as the Edinburgh claudication questionnaire proved inaccurate in 41% of cases seen.[3] It has been suggested that an automated oscillometric device commonly used for BP measurement is sufficiently accurate; a conclusion rejected in a comparative study by Hamel et al. [18,19] and not supported by NICE guidelines.[16] Adding a one-minute exercise test to conventional Doppler ABPI determination may marginally improve diagnosis.[20] Toe-finger photoplethysmography using low-cost infrared sensors, as are widely employed to measure digital oxygen saturation, is reported to be a fast and accurate alternative to Doppler APBI.[21] Another real-time technique under investigation is near-infrared spectroscopy.[7] More information is probably needed before one can safely recommend abandoning the current ‘gold standard’ of Doppler ABPI.

There is no universal agreement as to what should be considered a ‘normal’ ABPI result. Consequently, the authors decided that any value ≤1.0 should be deemed suspicious and warrant possible referral. This is at variance with the American Diabetes Association who quote the normal range of ABPI as 0.91–1.3.[22] We chose a higher value because results can be affected by variations in technique, gender, respiration, diabetes and hyper/hypotension and did not wish to risk misclassification in the current litigious climate. Other researchers also use 1.0 as a cut-off value which appears to correlate with the results of angiography or colour Duplex studies.[6] It must be emphasised that, whilst a low ABPI (e.g. < 0.6) is generally specific for peripheral arterial disease, the sensitivity of the test is less good, especially in the elderly and patients with diabetes.[7] Thus, a normal or high result (>1.4 suggests non-compressible, atherosclerotic arteries) in the presence of suspicious clinical features may still necessitate vascular referral.

## Limitations of study

This study involves a small, heterogeneous group together with limited and incomplete follow-up. Also, more diligent history taking and examination may have pointed to peripheral arterial disease, and determined the need for referral without ABPI measurement. However, we believe our findings still have value because they reflect real life experience amongst a diverse population with often multiple health issues.

## Governance

This was an observational study of routine practice overseen by senior practice staff for which ethical committee approval was not considered necessary.
